# Clinical Characteristics, Treatment and Prognosis of Primary Tracheal Adenoid Cystic Carcinoma: A Multicenter Retrospective Study

**DOI:** 10.1002/cam4.70877

**Published:** 2025-04-18

**Authors:** Yi Luo, Jun Teng, Zhina Wang, Qinyan Hong, Hang Zou, Lei Li, Nan Zhang, Hongwu Wang

**Affiliations:** ^1^ Respiratory Disease Center, Dongzhimen Hospital Beijing University of Chinese Medicine Tongzhou Beijing China; ^2^ Beijing University of Chinese Medicine Chaoyang Beijing China; ^3^ Department of Oncology Beijing Emergency General Hospital Chaoyang Beijing China; ^4^ Department of Pulmonary and Critical Care Medicine II Beijing Emergency General Hospital Chaoyang Beijing China

**Keywords:** lymph node status, prognosis, surgery, targeted therapy, tracheal adenoid cystic carcinoma

## Abstract

**Background:**

Tracheal adenoid cystic carcinoma (TACC) is a rare salivary gland malignant tumor. Previous studies mainly focused on surgery, radiation, and chemotherapy. The purpose of this study is to describe more clinical characteristics, treatments, and overall survival (OS) of TACC.

**Methods:**

Retrospectively analyzed TACC patients from two medical institutions and the SEER database from January 2010 to December 2021. Survival curves were drawn using the Kaplan–Meier method, and the effects of prognosis were analyzed by multivariate COX regression and AFT. The endpoint of the study was overall survival (OS).

**Results:**

One hundred fifty TACC patients were enrolled (DZM 11, EG 64, SEER 75), and the 5‐ and 10‐year survival rate was 70.62% and 35.80%, with a median survival time of 98 months. Lymph node status (yes) is an independent risk factor for TACC (HR = 3.020, 95% CI = 1.419–6.426, *p* = 0.004), and surgery is an independent protective factor (HR = 0.293, 95% CI = 0.146–0.587, *p* = 0.001). The AFT yielded similar results. In subgroup analysis of 63 non‐surgical patients, lymph node status (Yes) (HR = 3.511, 95% CI = 1.498–8.229, *p* = 0.004), and tumor longitudinal diameter range (TLDR) > 1 (HR = 2.975, 95% CI = 1.360–6.506, *p* = 0.006) are independent risk factors, while Targeted Therapy (HR = 0.248, 95% CI = 0.096–0.637, *p* = 0.004) is an independent protective factor.

**Conclusion:**

Lymph node status and TLDR are prognostic factors of TACC. Surgery is associated with prolonged survival of TACC. Targeted therapy may be associated with improved survival among non‐surgical TACC patients.

**Trial Registration:** ChiCTR2400083551

## 1 Introduction

Tracheal adenoid cystic carcinoma (TACC) is a rare salivary gland malignant tumor, accounting for 10%–20% of primary tracheal malignant tumors, second only to tracheal squamous carcinoma [1, 2]. TACC originates from the submucosal glands of the trachea; although it is an indolent low‐grade adenocarcinoma, it has a high tendency for perineural invasion, local recurrence, and distant metastasis [3, 4]. Common clinical symptoms of TACC include dyspnea, cough, and hemoptysis; due to its nonspecific clinical symptoms and imaging features, TACC is highly susceptible to misdiagnosis and usually has a diagnostic delay of approximately 1 year [5].

Surgery is one of the main treatments for TACC [6] and may contribute to better survival [7, 8, 9], but some patients are unable to undergo surgical treatment or achieve no residual tumor (R0) resection [10]. For those who cannot undergo surgical treatment or cannot achieve R0 resection, radiation and chemotherapy are the first choices, but their efficacy is still controversial [11, 12]. Previous studies on TACC have primarily focused on surgery, radiation, and chemotherapy, with limited exploration of targeted therapies and interventional bronchoscopy. Moreover, these studies have often been constrained by small sample sizes or single‐center designs. Based on this, the purpose of this study is to retrospectively review the information of TACC patients in two Chinese medical institutions from 2010 to 2021, as well as the corresponding data from the Surveillance, Epidemiology, and End Results (SEER) during the same period, to describe the clinical characteristics, treatments, and survival outcomes, including the evaluation of targeted therapies and interventional bronchoscopy, thereby contributing new insights to the existing literature.

## 2 Methods

### 2.1 Study Design and Patients

The Chinese cohort included 89 patients with TACC treated at Dongzhimen Hospital of Beijing University of Chinese Medicine (DZM) and the Emergency General Hospital (EG) from January 2010 to December 2021. The exclusion criteria were as follows: (1) age < 18 years; (2) ACC originated from other sites and metastasized or invaded the trachea; (3) patients with concurrent other pathological types of tumors; (4) missing survival data.

The SERR cohort included 96 patients with TACC registered from January 2010 to December 2021. The exclusion criteria were as follows: (1) age < 18 years; (2) ACC originated from other sites and metastasized or invaded the trachea; (3) patients with concurrent other pathological types of tumors; (4) patients with a recorded survival time of less than 1 month.

**FIGURE 1:**
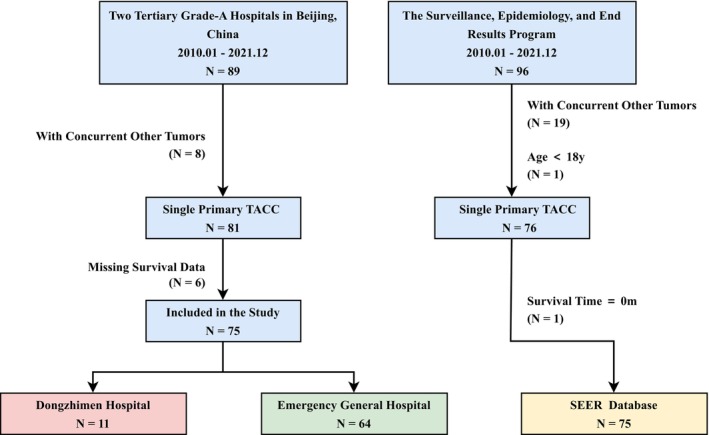
Flowchart of inclusion and exclusion criteria.

The study ultimately enrolled a total of 150 patients (Figure 1). This retrospective study was approved by the Ethics Committee of DZM (approval number:) and EG(approval number:). The Ethics Committee waived the requirement for informed consent for some participants. The surviving patients provided written informed consent. As the SEER database does not contain individually identifiable information and the data are publicly accessible, ethical approval was not required for this portion of the study.

### 2.2 Data and Definitions

Demographic information, initial symptoms, family history of tumors, and history of primary treatment (surgery, radiation, chemotherapy, immunotherapy, targeted therapy, photodynamic therapy (PDT), stent placement) of the patients were extracted from the electronic medical record systems of the two centers. The outcome measure for this study is OS, which is defined as the time from the pathological diagnosis of TACC to death or the last follow‐up. The dates of pathological diagnosis were retrieved from the electronic medical record systems of the two centers. The dates of death were obtained through medical record registration, telephone follow‐up, and inquiries with the Chinese Center for Disease Control and Prevention. The last follow‐up date for this study was December 31, 2023.

Due to the low incidence of TACC, there is currently no unified staging system for this tumor. In the absence of a specific staging system, the study refers to the assessment method for malignant airway stenosis proposed by Chinese experts to evaluate the tumor [13, 14]. This method provides a practical framework for the evaluation and treatment of malignant airway stenosis, including various airway tumors. Specifically, the tumor longitudinal diameter range (TLDR), tumor transverse diameter range (TTDR), and degree of airway stenosis (DSA) were assessed by chest computed tomography (CT) and bronchoscopy. Additionally, the lymph node status and distant metastasis of the tumor were recorded. The trachea was divided into three equal segments for the purpose of assessing TTDR; the upper 1/3 was designated as Zone I, the middle 1/3 as Zone II, and the lower 1/3 as Zone III. TTDR was classified into T1 (involving only intraluminal, mural, or extramural) and T2 (mixed).

In SEER database, the extracted information includes age, gender tumor size, lymph node status, tumor metastasis, treatment information (surgery, radiotherapy, chemotherapy), survival time, and survival status.

### 2.3 Statistical Analyses

Continuous variables are described as mean ± SD, and analysis is performed using equal variance *t*‐test or Mann–Whitney *U* test based on their distribution characteristics. Count data are presented as frequencies and percentages, and chi‐squared test or Fisher's exact probability method is selected based on sample size.

Kaplan–Meier survival curves were used to compare survival information from the Chinese and SEER cohorts. Group comparisons of survival rates between groups based on whether patients received surgery, radiotherapy, or chemotherapy were performed using the log‐rank test. Clinical factors influencing patients' OS were analyzed using a single‐factor Cox proportional hazards regression model. Factors with *p* < 0.1 in the single‐factor analysis were included in a multi‐factor Cox proportional hazards regression model to further analyze independent prognostic factors affecting survival. Because sex did not meet the proportional hazards assumption, we used an AFT model, which does not rely on this assumption, for additional validation. The AFT model converts parameter estimates to time ratio (TR) estimates to explain the effect of covariates on the time scale. TR > 1 indicates that a covariate is positively associated with survival time (prolongs median survival), whereas TR < 1 indicates that a covariate is negatively associated with survival time (shortens median survival) [15, 16].

All analyses were performed using R software version 4.4.0 (https://www.r‐project.org/) and RStudio version 2024.04.0 + 735 (https://posit.co/). The R packages used included readr, dplyr, survival, survminer, glmnet, tableone, ggplot2, interactions, and car. All tests were two‐tailed, and *p* < 0.05 was considered statistically significant.

## 3 Results

### 3.1 Patient Characteristics

A total of 150 patients were enrolled in the study (Figure 1) (Medical InstitutionA 11, Medical InstitutionB 64, SEER 75). The Chinese cohort included 75 TACC patients (Table 1). The mean age of the patients was 48.47 ± 12.86 years, and 45.3% were female. The most common initial symptoms were cough and sputum (48%) and dyspnea (36%). Among them, 42.7% of the patients had a history of smoking, and 17.3% had a family cancer history. The proportion of patients with TLDR involving more than one Zone (TLDR > 1) was 41.3%. 66.7% of patients with TTDR were T2 type, and a majority of patients (65.3%) DSA > 50%. 28% of patients had lymph node metastasis, and 45.3% had tumor metastasis. In the Chinese cohort, 16% of the patients underwent surgery, 57.3% received radiotherapy, 38.7% received chemotherapy, 5.3% received immunotherapy, 32% received targeted therapy, 18.7% received photodynamic therapy, and 46.7% had airway stent placement.

**TABLE 1:** Clinical features and treatment of the patients.

Characteristics	DZM and EG (*N* = 75)	SEER (*N* = 75)	*p* value
Age, year (mean ± SD)	48.47 ± 12.86	55.31 ± 15.81	0.004
Sex (%)			
Female	34 (45.3)	45 (60.0)	0.102
Male	41 (54.7)	30 (40.0)	
Initial symptoms (%)			
Cough and sputum	36 (48.0)		
Dyspnea	27 (36.0)		
Hemoptysis	5 (6.7)		
Others	7 (9.3)		
Smoking history (%)			
Yes	32 (42.7)		
No	43 (57.3)		
Family cancer history (%)			
Yes	13 (17.3)		
No	62 (82.7)		
TLDR (%)^a^			
I	44 (58.7)		
II	25 (33.3)		
III	6 (8.0)		
TTDR^b^ (%)			
T1	25 (33.3)		
T2	50 (66.7)		
DAS^c^ (%)			
1	6 (8.0)		
2	20 (26.7)		
3	25 (33.3)		
4	21 (28.0)		
5	3 (4.0)		
Tumor size, mm (mean ± SD)			
Known		31.05 ± 13.40	
Unknown		11 (14.7)	
Lymph node status (%)			
N_+_	21 (28.0)	7 (9.3)	0.000
N_−_	39 (52.0)	16 (21.3)	
N_×_	15 (20.0)	52 (69.3)	
Tumor metastasis (%)			
Yes	34 (45.3)	11 (14.7)	0.000
No/Unknown	41 (54.7)	64 (85.3)	
Surgery (%)			
Yes	12 (16.0)	52 (69.3)	0.000
No	63 (84.0)	23 (30.7)	
Radiation (%)			
Yes	43 (57.3)	53 (70.7)	0.126
N/U^d^	32 (42.7)	22 (29.3)	
Chemotherapy (%)			
Yes	29 (38.7)	21 (28.0)	0.225
N/U	46 (61.3)	54 (72.0)	
Immunotherapy (%)			
Yes	4 (5.3)		
No	71 (94.7)		
Targeted therapy (%)			
Yes	24 (32.0)		
No	51 (68.0)		
PDT (%)			
Yes	14 (18.7)		
No	61 (81.3)		
Stent placement (%)			
Yes	35 (46.7)		
No	40 (53.3)		

^a^ Tumor longitudinal diameter range.

^b^ Tumor transverse diameter range.

^c^ Degree of airway stenosis.

^d^ No/Unknown.

The SEER cohort included 75 patients (Table 1), with a mean age of 55.31 ± 15.81 years, and 60% were female. Among the patients with available data, the mean tumor size was 31.05 ± 13.40 mm, with 9.3% of patients having lymph node metastasis and 14.7% having distant metastasis. In the SEER cohort, 69.3% of patients underwent surgery, 70.7% received radiotherapy, and 28% were treated with chemotherapy.

Among the total of 150 TACC patients, 42.7% underwent surgery, 64.0% received radiotherapy, and 33.3% received chemotherapy (Table 2). While 38.7% of patients received multiple modalities of treatment, including combinations of surgery, radiation, and chemotherapy. Specifically, 29.3% of patients received both surgery and radiation therapy, 9.3% received surgery combined with chemotherapy, and 8.0% received surgery, radiation, and chemotherapy.

**TABLE 2:** Multiple modalities of treatment.

Treatment	DZM and EG (*N* = 75)	SEER (*N* = 75)	ALL (*N* = 150)
Surgery (%)	12 (8.0)	52 (34.7)	64 (42.7)
Radiation (%)	43 (28.7)	53 (35.3)	96 (64.0)
Chemotherapy (%)	29 (19.3)	21 (14.0)	50 (33.3)
Surgery and radiation (%)	7 (4.7)	37 (24.7)	44 (29.3)
Surgery and chemotherapy (%)	5 (3.3)	9 (6.0)	14 (9.3)
Surgery and radiation and chemotherapy (%)	4 (2.7)	8 (5.3)	12 (8.0)

### 3.2 Survival Analysis

**FIGURE 2:**
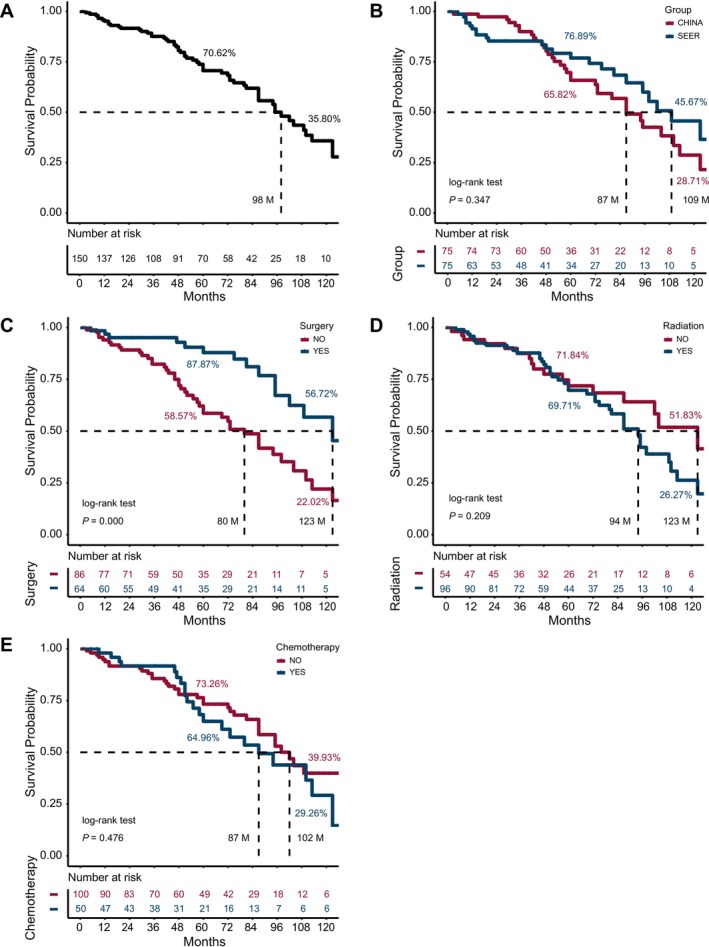
Kaplan–Meier Survival analyses for TACC patient. (A) Survival analysis for 150 TACC patients, indicating comparative 5‐ and 10‐year overall survival (OS) rates and median survival time in months (M). (B) Group by Chinese and SEER cohort. (C) Group by surgery. (D) Group by radiation. (E) Group by chemotherapy.

The 5‐, and 10‐year survival rate for the 150 TACC patients was 70.62%, and 35.80%, with a median survival time of 98 months (Figure 2A). The Chinese cohort had a 5‐year survival rate of 65.82% and a 10‐year survival rate of 28.71%, with a median survival time of 87 months; the SEER cohort had a 5‐year survival rate of 76.89% and a 10‐year survival rate of 45.67% with a median survival time of 109 months, and there were no significant differences between the two cohorts (*p* = 0.347) (Figure 2B). Compared with patients who did not undergo surgery, those who underwent surgery had improved 5‐year and 10‐year survival rates, and prolonged median survival time, with significant differences in survival rates between the groups (*p* < 0.05) (Figure 2C). Compared with patients who did not receive radiotherapy and chemotherapy, there were no significant differences in survival rates for patients who received radiotherapy (*p* = 0.209) and chemotherapy (*p* = 0.476) (Figure 2D,E).

### 3.3 Factors Associated With the Prognosis of TACC

The results of the univariate Cox regression survival analysis indicated that lymph node status and surgery may be significant prognostic factors for TACC (*p* < 0.05) (Table 3). In multivariate Cox Regression Survival Analysis, we further considered lymph node status (unknown), and tumor metastasis with *p* < 0.1. The analysis revealed that lymph node status (yes) is an independent risk factor for TACC (HR = 3.020, 95% CI = 1.419–6.426, *p* = 0.004), while surgery is an independent protective factor (HR = 0.293, 95% CI = 0.146–0.587, *p* = 0.001). To corroborate these findings, we employed the AFT model as a supplementary validation, yielding similar results. Lymph node status (Yes) was associated with a reduction in median survival time (TR = 0.591, 95% CI = 0.380–0.918, *p* = 0.019), whereas surgery was associated with an extension of median survival time (TR = 2.224, 95% CI = 1.402–3.528, *p* = 0.001).

**TABLE 3:** Cox regression survival analysis and AFT analysis (*N* = 150).

Features	Univariate Cox		Multivariate Cox		Multivariate AFT^c^	
	HR (95% CI)	*p*	HR (95% CI)	*p*	TR (95% CI)	*p*
Age^a^, 1 year	1.004 (0.986–1.023)	0.644			0.993 (0.982–1.004)	0.199
Cohort, SEER	0.777 (0.460–1.312)	0.346			0.885 (0.526–1.488)	0.644
Sex^b^, male	0.969 (0.580–1.618)	0.903			1.072 (0.788–1.459)	0.658
Tumor metastasis, yes	1.841 (1.097–3.090)	0.021	0.851 (0.437–1.657)	0.635	0.980 (0.642–1.496)	0.927
Lymph node status, unknown	1.501 (0.810–2.783)	0.197	2.183 (1.149–4.146)	0.017	0.661 (0.411–1.062)	0.087
Lymph node status, yes	2.755 (1.398–5.427)	0.003	3.020 (1.419–6.426)	0.004	0.591 (0.380–0.918)	0.019
Surgery, yes	0.365 (0.202–0.659)	0.001	0.293 (0.146–0.587)	0.001	2.224 (1.402–3.528)	0.001
Radiation, yes	1.431 (0.819–2.502)	0.209			0.875 (0.612–1.250)	0.462
Chemotherapy, yes	1.215 (0.716–2.064)	0.470			1.091 (0.777–1.532)	0.615

^a^ Continuous variable.

^b^ Fails the proportional hazards assumption test, *p* = 0.018.

^c^ The Weibull distribution was selected based on the lowest AIC among the lognormal, Weibull, and exponential distributions.

### 3.4 Subgroup Analysis of Non‐Surgical Patients in the Chinese Cohort

In subgroup analysis, we conducted a further analysis of 63 TACC patients in the Chinese cohort who did not receive surgery, aiming to explore effective treatment beyond surgery. Through univariate Cox Regression Survival Analysis, we selected four variables with *p* < 0.1, TLDR > 1, lymph node status (unknown), lymph node status (yes), and Targeted Therapy, for inclusion in the multivariate Cox Regression Survival Analysis (Table 4). The results revealed that lymph node status (yes) (HR = 3.511, 95% CI = 1.498–8.229, *p* = 0.004), lymph node status (unknown) (HR = 4.3, 95% CI = 1.511–12.241, *p* = 0.006), and TLDR > 1 (HR = 2.975, 95% CI = 1.360–6.506, *p* = 0.006) are independent risk factors for non‐surgical patients, while Targeted Therapy (HR = 0.248, 95% CI = 0.096–0.637, *p* = 0.004) is an independent protective factor.

**TABLE 4:** Cox Regression Survival Analysis of non‐surgical patients in the Chinese cohort (*N* = 63).

Features	Univariate Cox^a^		Multivariate Cox	
	HR (95% CI)	*p*	HR (95% CI)	*p*
Smoking history, yes	1.100 (0.537–2.252)	0.795		
Family cancer history, yes	1.347 (0.569–3.189)	0.498		
TLDR^b^, > 1	0.476 (0.230–0.986)	0.046	2.975 (1.360–6.506)	0.006
TTDR^c^, T2	1.250 (0.587–2.659)	0.563		
DAS^d^, > 50%	0.747 (0.356–1.567)	0.440		
Lymph node status, unknown	2.486 (0.926–6.678)	0.071	4.3 (1.511–12.241)	0.006
Lymph node status, yes	2.421 (1.099–5.332)	0.028	3.511 (1.498–8.229)	0.004
Immunotherapy, yes	0.935 (0.277–3.157)	0.913		
Targeted Therapy, yes	0.483 (0.208–1.118)	0.089	0.248 (0.096–0.637)	0.004
PDT, yes	0.613 (0.142–2.639)	0.511		
Stent placement, yes	1.233 (0.614–2.474)	0.556		

^a^ Meets the proportional hazards assumption test, all *p* > 0.05.

^b^ Tumor longitudinal diameter range.

^c^ Tumor transverse diameter range.

^d^ Degree of airway stenosis.

## 4 Discussion

This study reviewed the clinical characteristics of TACC patients from two medical institutions in China and the SEER database, and compared the long‐term prognosis of different treatment methods, which not only included traditional treatment such as surgery, radiation, and chemotherapy, but also systemic treatment such as immunotherapy and targeted therapy, as well as interventional bronchoscopy such as PDT and stent placement. Our study found that surgery significantly prolonged the survival of TACC patients (Figure 2C), and lymph node status was identified as an independent risk factor for TACC (Table 3). In subgroup analysis focusing on Chinese patients who did not undergo surgery, we further explored potential predictive factors and treatment. Lymph node status remains a predictive factor, TLDR may also be associated with prognosis, and the potential effects of targeted therapy warrant further validation (Table 4).

In this study, we found significant differences in age between the Chinese cohort and the SEER cohort (Table 1), but overall, the distribution of age and gender was consistent with previous literature [6, 17]. Furthermore, no significant correlation was found between age and gender and the OS of TACC patients (Table 3). Our study did not include participants under the age of 18 because the two medical institutions involved in the study had not treated any pediatric TACC patients. TACC is extremely rare in pediatrics. Dumitru and Balica reported a case of subglottic tracheal adenoid cystic carcinoma in a 16‐year‐old female [18]. Wei Liu and Xiaohong Chen reported a case of a 15‐year‐old patient with laryngeal adenoid cystic carcinoma (ACC) [19]. Other reports of pediatric ACC are more commonly seen in the parotid and submandibular glands [20, 21]. Compared with adult patients, the treatment of pediatric TACC poses greater challenges. The choice of treatment should be made with caution, and the long‐term side effects, such as growth retardation, hormonal dysfunction, and cognitive impairment should be fully considered [22, 23].

In the Chinese cohort, cough, dyspnea, and hemoptysis were the most common initial symptoms of TACC patients (Table 1). Previous studies have indicated that tumor size [24], location [25], and extent of invasion [9] are important factors affecting prognosis. In this study, we assessed tumor characteristics using three indicators: TLDR, TTDR, and DAS. The results showed that TLDR > 1 was an adverse prognostic factor for non‐surgical TACC patients, while TTDR and DAS were not significantly correlated with prognosis (Table 4). A previous study found that CT longitudinal length of tumors showed satisfactory discrimination for TACC [26]. TACC is characterized by a diffuse infiltrative morphology with a long segment of submucosal involvement [27]. For patients not suitable for surgery, assessing tumor size is challenging. TLDR, as a relatively simple and easily assessable indicator in radiological and bronchoscopic evaluation, deserves further attention in the assessment of TACC characteristics.

Lymph node metastasis is generally considered a prognostic risk factor for malignant tumors. However, previous studies have reported conflicting results regarding the impact of lymph node metastasis on the prognosis of TACC, with some studies showing no significant correlation with prognosis [24, 28]. Yang et al.'s study of 263 patients with tracheobronchial ACC based on the SEER database showed that lymph node ratio and total lymph node count were prognostic factors for survival of ACC patients [4]. In our study, the results indicate a significant correlation between TACC prognosis and lymph node metastasis in both the overall data (Table 3) and the subgroup analysis (Table 4). However, given the significant presence of lymph node status (unknown) in the analysis, we believe that the study results should be cautiously interpreted. Overall, assessing lymph node metastasis, when conditions permit, is valuable for the prognosis of TACC.

Surgery is currently recognized as a treatment for TACC that can significantly improve the prognosis. Consistent with the results of previous studies [7, 9, 29], surgery can significantly prolong the survival time of TACC patients in our study, which is an independent prognostic factor (Table 3). It is also difficult to achieve R0 resection [10], but there was little correlation between positive margins and recurrence [30], and there was no significant difference in prognosis between patients with positive and negative margins [9, 11]. Studies have shown that the long‐term survival rate and disease‐free survival rate of complex airway resection are similar to those of standard surgery, but it will lead to early postoperative complications and mortality [29]. Therefore, it is reasonable to explore other systemic and local treatment options.

Radiotherapy is an important treatment for TACC patients who are not candidates for surgical resection or have undergone incomplete resection. However, the efficacy of both definitive and postoperative radiotherapy remains a subject of debate [11, 12, 29, 31]. In our study, radiotherapy did not significantly impact the survival of TACC patients (Table 3, Figure 2D). Some studies have suggested that the prognosis of TACC is related to radiotherapy dose [32, 33]. A study proposes that for radiotherapy, irradiation with C^12^ may have advantages over conventional radiation in terms of local and distant metastasis control [8]. In addition, for patients who cannot tolerate external beam radiotherapy, brachytherapy—such as ^125^I seed implantation—has shown promising short‐term complete response rates and 2‐year recurrence‐free survival rates [34]. The indications, dosage, and methods of radiotherapy for TACC warrant further exploration.

Systemic therapy is an important treatment modality for malignant tumors. For TACC patients who are ineligible for surgical treatment and those with postoperative recurrence and metastasis, the exploration of effective systemic treatment options is highly relevant. Currently, chemotherapy is mainly used for palliative treatment of metastatic and recurrent TACC, primarily with platinum‐based mono‐ or combination therapy regimens [35], but there is no evidence to prove the efficacy of chemotherapy in treating TACC. PD‐L1 is either poorly expressed or not expressed in ACC or tumor‐infiltrating lymphocytes [36, 37], suggesting that the efficacy of immunotherapy may be limited. In a randomized, Phase II clinical study of pembrolizumab with or without radiotherapy for recurrent or metastatic ACC, immunotherapy did not yield satisfactory results, but researchers believe that further exploration of the synergistic effects between radiotherapy and immunotherapy is valuable [38].

Only a few cases of targeted therapy in TACC have been reported [39, 40]. Our study conducted a subgroup analysis of 63 non‐surgical patients; the results indicated that neither chemotherapy nor immunotherapy conferred a survival benefit, but targeted therapy may have potential benefits (Table 4). In our study, 24 patients received intratumoral antineoplastic therapy with Endostar under bronchoscopy for targeted therapy. Endostar is a novel recombinant human endostatin that exerts an antiangiogenic effect via blocking VEGF‐induced tyrosine phosphorylation of KDR/Flk‐1 of endothelial cells [41]. Angiogenesis plays a critical role in the progression of ACC, as it provides the necessary oxygen and nutrients to the tumor, promoting its growth and metastasis. Anti‐angiogenic therapy, particularly drugs targeting vascular endothelial growth factor receptor (VEGFR), should theoretically be effective against ACC. However, current clinical studies have not shown expected results with anti‐VEGFR drugs in the treatment of ACC, including Lenvatinib [42, 43, 44], Axitinib [45], and Sorafenib [46, 47]. Targeted therapies against MYB and its downstream oncogenic activation pathways are still under exploration. Retinoic acid agonists inhibited tumor growth in vivo in ACC patient‐derived xenograft models and decreased MYB binding at translocated enhancers, thereby potentially diminishing the MYB positive feedback loop driving ACC [48]. However, the Phase II clinical trial of all‐trans retinoic acid (ATRA) in the treatment of advanced ACC did not yield satisfactory results [49]. Previous studies focused on systemic treatments; this study suggests that perhaps intratumoral antineoplastic therapy with targeted drug under bronchoscopy is a direction worth further exploration.

In the subgroup analysis of our study, there was no significant correlation between PDT and stent placement with the prognosis of non‐surgical TACC patients (Table 4). However, the outcome focused on in this study was Overall Survival (OS), without assessing short‐term efficacy. On the one hand, interventional bronchoscopy may provide effective palliative treatment for TACC, and on the other hand, it can be used as an auxiliary means to provide opportunities for surgery, radiotherapy, and systemic treatment [50, 51]. Previous studies have indicated that PDT can reduce the tumor burden of TACC, alleviate clinical symptoms, and improve the quality of life [52, 53], making it a promising treatment that requires more clinical practice. Stent placement can also effectively relieve airway stenosis, and small‐sample studies have shown that interventional bronchoscopy including stent implantation for unresectable primary tracheal tumors does not seem to be burdened by significant complications and may provide survival benefits for TACC patients [50]. Additionally, stenting is a safe and effective treatment method for fibrotic airway stenosis induced by radiotherapy in patients with TACC [54]. At present, our study did not find any adverse effect of PDT and stent implantation on the long‐term prognosis of TACC (Table 4).

There are still some limitations in this study. First, as a multicenter clinical study, the study did not take into account race, region, and socioeconomic factors, which may have some influence on prognosis. Second, the evaluation methods of tumor characteristics and treatments were not entirely consistent between the Chinese cohort and the SEER cohort; therefore, we are unable to make a comprehensive evaluation of some items. Additionally, due to the limitations of the data sources, we were unable to obtain the specific data on cancer‐specific survival and progression‐free survival. Finally, the study focused mainly on whether to receive treatment or not, and did not fully consider the effects of treatment procedure, radiation dose, drug dose, frequency of treatment, and combined therapies. Therefore, although some positive results were obtained, we believe that the results should be assessed with caution. Despite these limitations, this study first‐time focuses on the treatment and prognosis of non‐surgical TACC patients, and provides a reference for future clinical studies of TACC.

## 5 Conclusions

In this multicenter retrospective study, we evaluated the clinical characteristics, treatment, and prognosis of TACC. Lymph node status was identified as an independent risk factor for the prognosis of TACC; surgery was significantly associated with prolonged OS. For those non‐surgical TACC patients, both TLDR and lymph node status were significant independent prognostic factors, and targeted therapy may be associated with extended OS. The identification of TLDR as an independent prognostic factor offers a new perspective for clinical decision‐making. Furthermore, the potential benefits of targeted therapy among non‐surgical patients may represent a promising direction for further exploration.

## Author Contributions

**Yi Luo:** formal analysis, methodology, software, writing original draft. **Jun Teng:** formal analysis, founding acquisition, software, writing original draft. **Zhina Wang:** investigation, data curation, validation. **Qinyan Hong:** investigation, data curation, visualization. **Hang Zou:** project administration, validation. **Lei Li:** project administration, data curation. **Nan Zhang:** conceptualization, supervision, resources. **Hongwu Wang:** conceptualization, supervision, founding acquisition, resources. The review and editing of the manuscript involved all authors, who also gave their approval for the final version.

## Ethics Statement

This study was approved by the Ethics Committees of Dongzhimen Hospital, Beijing University of Chinese Medicine (No. 2024DZMEC‐039‐02), and Emergency General Hospital (No. K24‐24), adhering to the ethical standards of the Helsinki Declaration. The SEER database does not contain individually identifiable information and the data are publicly accessible, ethical approval was not required for this portion of the study.

## Consent

The Ethics Committee waived some requirements for informed consent. Surviving patients signed written informed consent.

## Conflicts of Interest

The authors declare no conflicts of interest.

## Data Availability

The data that support the findings of this study are available from the corresponding author upon reasonable request.
